# Neuroprotective Effects of CXCR2 Antagonist SB332235 on Traumatic Brain Injury Through Suppressing NLRP3 Inflammasome

**DOI:** 10.1007/s11064-023-04021-8

**Published:** 2023-09-13

**Authors:** Ke Zhao, Xinkui Zhou, Mengyuan Chen, Lingshan Gou, Daoqi Mei, Chao Gao, Shuai Zhao, Shuying Luo, Xiaona Wang, Tao Tan, Yaodong Zhang

**Affiliations:** 1 Children’s Hospital Affiliated to Zhengzhou University, Henan Children’s Hospital, Zhengzhou Children’s Hospital, Henan Key Laboratory of Children’s Genetics and Metabolic Diseases, Henan Children’s Neurodevelopment Engineering Research Center, Zhengzhou, China; 2 Center for Genetic Medicine, Xuzhou Maternity and Child Health Care Hospital Affiliated to Xuzhou Medical University, Xuzhou, China; 3 Department of Neurology, Children’s Hospital Affiliated to Zhengzhou University, Zhengzhou, China; 4 Department of Rehabilitation, Children’s Hospital Affiliated to Zhengzhou University, Zhengzhou, China; 5 Oujiang Laboratory (Zhejiang Lab for Regenerative Medicine, Vision and Brain Health), Key Laboratory of Alzheimer’s Disease of Zhejiang Province, Institute of Aging, Wenzhou Medical University, Wenzhou, Zhejiang China

**Keywords:** Traumatic brain injury, CXCR2 antagonist, SB332235, NLRP3 inflammasome, Anti-inflammation, Microglia

## Abstract

The inflammatory process mediated by nucleotide-binding oligomerization domain (NOD)-like receptor family pyrin domain comprising 3 (NLRP3) inflammasome plays a predominant role in the neurological dysfunction following traumatic brain injury (TBI). SB332235, a highly selective antagonist of chemokine receptor 2 (CXCR2), has been demonstrated to exhibit anti-inflammatory properties and improve neurological outcomes in the central nervous system. We aimed to determine the neuroprotective effects of SB332235 in the acute phase after TBI in mice and to elucidate its underlying mechanisms. Male C57BL/6J animals were exposed to a controlled cortical impact, then received 4 doses of SB332235, with the first dose administered at 30 min after TBI, followed by additional doses at 6, 24, and 30 h. Neurological defects were assessed by the modified neurological severity score, while the motor function was evaluated using the beam balance and open field tests. Cognitive performance was evaluated using the novel object recognition test. Brain tissues were collected for pathological, Western blot, and immunohistochemical analyses. The results showed that SB332235 significantly ameliorated TBI-induced deficits, including motor and cognitive impairments. SB332235 administration suppressed expression of both CXCL1 and CXCR2 in TBI. Moreover, SB332235 substantially mitigated the augmented expression levels and activation of the NLRP3 inflammasome within the peri-contusional cortex induced by TBI. This was accompanied by the blocking of subsequent production of pro-inflammatory cytokines. Additionally, SB332235 hindered microglial activity induced by TBI. These findings confirmed the neuroprotective effects of SB332235 against TBI, and the involved mechanisms were in part due to the suppression of NLRP3 inflammasome activity. This study suggests that SB332235 may act as an anti-inflammatory agent to improve functional outcomes in brain injury when applied clinically.

**Supplementary Information:**

The online version contains supplementary material available at 10.1007/s11064-023-04021-8.

## Introduction

Traumatic brain injury (TBI) is a severe and common neurosurgical injury resulting from abrupt blunt impact, inadvertent skull penetration, or explosive blast overpressure that damages the brain [1, 2]. TBI is a life-threatening condition that is associated with high morbidity and mortality rate [3]. It has been reported that over 50 million people worldwide suffer from TBI each year, with some experiencing permanent disabilities [4, 5]. Nonetheless, effective therapeutic interventions to improve functional outcomes in TBI patients are currently unavailable.

TBI has an intricate pathophysiology that can be composed of primary and secondary injuries. Because the primary injury is generally regarded as an irrecoverable progression, current managements of TBI concentrates on secondary injury, particularly optimizing the neurological outcome [6]. It is well established that post-traumatic neuroinflammation responses play a significant role in the mechanism and neurological deficits [7, 8]. The overactivation of neuroinflammation is perceived as the fundamental cause of various complications that occur post-TBI, including motor dysfunction and cognitive defects [9]. Thus, anti‑inflammation treatment may be a beneficial option to mitigate secondary brain injuries and improve consequent disabilities following TBI [10].

Recent efforts have demonstrated that the activation of NOD-, LRR- and pyrin domain-containing protein 3 (NLRP3) inflammasome plays a pivotal role in the regulation of the TBI-induced neuroimmune response [11, 12]. TBI activates NLRP3 inflammasome resulting in the elevation of pro-inflammatory cytokine production [13, 14]. Moreover, converging evidence reveals that TBI results in NLRP3 inflammasome activation in microglia [12, 15, 16], which releases several key pro-inflammatory cytokines, such as Interleukin (IL)-1β, IL-6, Tumor Necrosis Factor-alpha (TNF-α) [14]. These findings manifest that the NLRP3-IL-1β signaling pathway plays a prominent role in TBI-induced neuroinflammation.

Considerable attention has been paid to chemokine receptor 2 (CXCR2), a G protein-coupled receptor regulated by CXC chemokines [17]. Robust evidence suggests that chemokine (CXC motif) ligand 1 (CXCL1) and its receptor, CXCR2, are involved in NLRP3 activation [18]. Studies have indicated that CXCR2 expression is enhanced in the cortex of patients and animal models after brain trauma [19–21]. Importantly, it has been established that CXCR2 involves in the activation of NLRP3 inflammasome and pro-inflammatory factors from microglia [18, 22]. The specific inhibitor of CXCR2, SB332235, has been shown to have neuroprotective pharmacological effects [17]. However, it is yet to be determined whether SB332235 can alleviate TBI-associated neurobehavioral outcomes by restraining the activation of NLRP3 inflammasome.

To address this question, mice were subjected to TBI and then exposed with the CXCR2 antagonist, SB332235. The neurobehavioral sequelae, tissue structure, and neuron viability of ipsilateral cortical tissues were assessed. Furthermore, the administration of SB332235 on NLRP3 inflammasome pathway and microglial activity were also explored. Overall, the findings of this study demonstrate the significant neuroprotective action of SB332235 on TBI, which is associated with its inhibition of NLRP3 inflammasome activity.

## Materials and Methods

### Animals

All animal procedures were approved by Zhengzhou University’s Animal Care and Use Committee and conducted by the National Institutes of Health Guidelines for the Care and Use of Laboratory Animals. Male C57BL/6J mice (aged 8 weeks and weighting 20–24 g) were housed in a temperature and humidity-controlled (22 ± 1 °C, 55 ± 3%) vivarium with a regular 12 h light/dark cycle. Food and water were supplied *ad libitum*. All efforts were undertaken to minimize animal suffering and the number of animals used.

### Experimental Groups and Drug Administration

A total of 64 mice were assigned to the following four groups (16 mice per group): Sham + saline group (Sham), Sham + SB332235 group (Sham + SB), TBI + saline group (TBI), and TBI + SB332235 group (TBI + SB). Under conventional anesthesia, mice in the TBI and TBI + SB groups received moderate TBI. Mice in the Sham + SB and TBI + SB groups received intraperitoneal administration of SB332235 (Tocris Bioscience, Bristol, UK, diluted in saline). The first dose was administered at 30 min post-TBI, followed by additional doses at 6, 24, and 30 h by following the previous study [23]. A dose of 1 mg/kg was chosen based on previous studies [17]. The Sham and TBI-exposed mice received an equivalent volume of normal saline.

### TBI Model

Moderate TBI models were established using the controlled cortical impact method described previously [24]. In Brief, mice were anesthetized with 1.5% isoflurane in oxygen and secured on a stereotaxic frame. The coronal and sagittal sutures were completely exposed, and a craniotomy was conducted with the entire dura intact. A 4 mm diameter hole was drilled into the left parietal cortex, located 2 mm posterior to the bregma and 2 mm lateral to the sagittal suture. TBI mice received an impact using an electric cortical contusion impactor (RWD Life Science, Shenzhen, China) with a 4 mm diameter tip at a velocity of 3.5 m/s, depth of 1 mm, and a dwell time of 400 ms. The skull was then sealed and the scalp incision was closed using intermittent sutures. Sham-operated mice received the same craniotomy without impingement. All procedures were conducted under aseptic conditions.

### Behavioral Testing

**Fig. 1:**
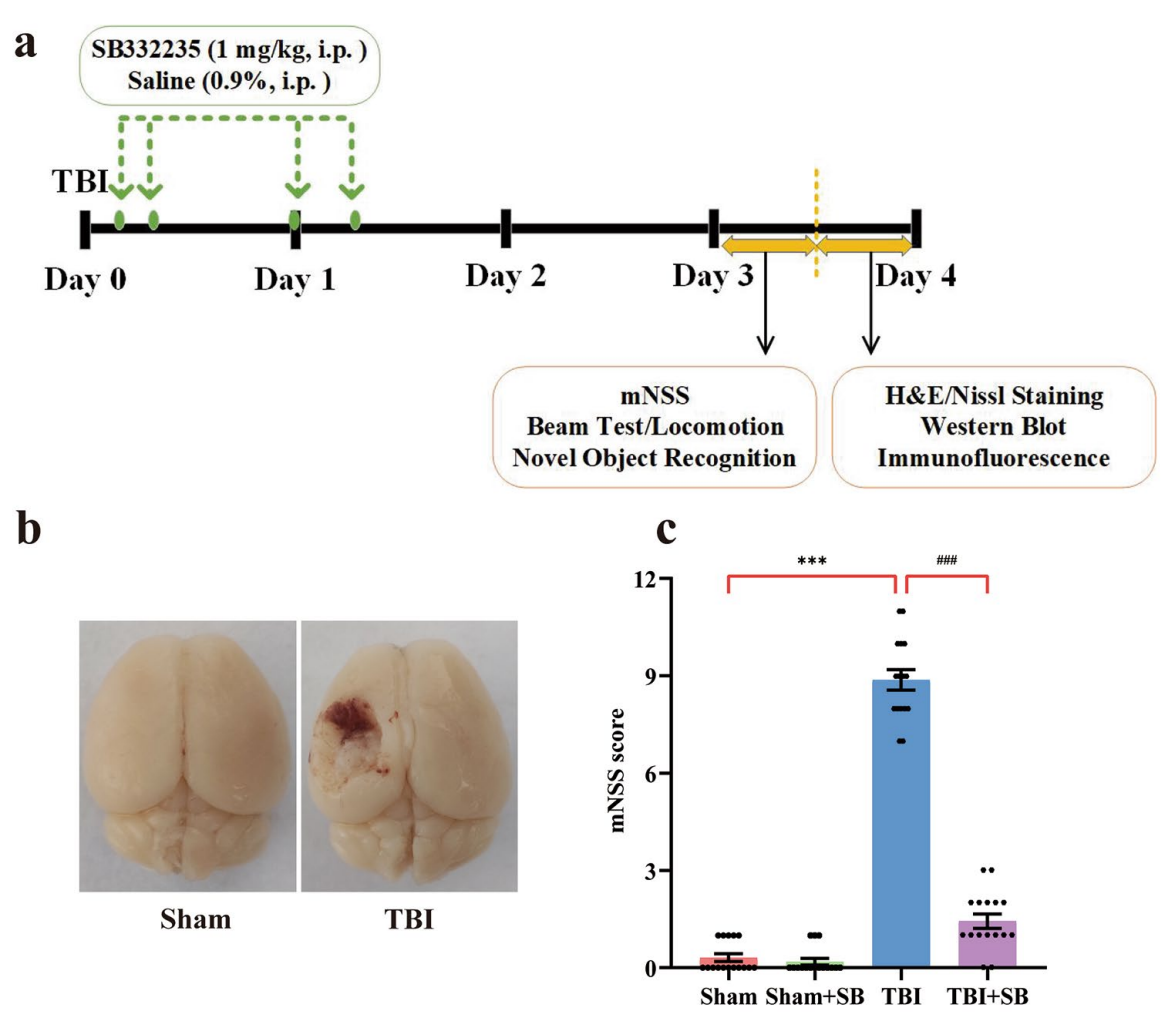
Effect of SB332235 on TBI-induced neurological deficits. (**a**) Schematic diagram of the experimental workflow. (**b**) Representative photographs of a normal (left) and damaged brain (right) after traumatic brain injury (TBI) caused by an electric cortical contusion impactor. (**c**) Neurological function was assessed using the modified neurological severity score (mNSS) at 3 days post-injury. Data are presented as mean ± SEM (*n* = 16 mice per group). Two-way ANOVA with Tukey’s *post hoc* test: ^***^p < 0.001 vs. Sham group; ^###^*p* < 0.001 vs. TBI group

Mice were subjected to a battery of behavioral tests at 3 days post-TBI, as recent studies suggest increased CXCR2 at this time point [21, 25]. After behavioral assessment, mice were sacrificed and the brains were quickly removed. The peri-contusional cortex was dissected according to the mouse atlas for immunohistochemical staining and Western blot experiments. The experimental design is presented in Fig. 1a.

### Modified Neurological Severity Scores

Modified neurological severity scores (mNSS) were adopted to evaluate the TBI-induced neurological impairments, as previously reported [26]. The mNSS is a comprehensive evaluation that contains motor, sensory, and reflex tests. The test is scored on a scale of 0 to 18 points, with 0 representing normal function and 18 representing maximal damage. Higher scores indicate greater levels of neurologic dysfunction.

### Beam-Balance and Beam-Walk Tests

To evaluate the gross and fine motor function, we employed beam-balance and beam-walk tasks [17]. For the beam-balance test, animals were placed on an elevated wooden beam (1.5 cm in width) and the duration was recorded. Mice were allowed a maximum of 60 s on the beam. The beam-walking task consisted of training mice to avoid bright light and loud white noise by traversing a narrow wooden beam (2.5 cm in width and 100 cm in length) and entering a darkened goal box at the other end. Performance was calculated based on the time taken to cross the beam. Mice were pre-trained on each task one day before the surgery to establish baseline performance. Three 60 s trials were conducted for each task on day 3 following TBI. Average scores for each mouse were used for statistical analysis.

### Open Field Test

The open-field test provides a synchronous assessment of locomotion and exploration, as reported before [27]. Briefly, mice were acclimatized for 30 min in the open field chamber (40 × 40 × 32 cm). Animals were then dropped into the open field chamber and allowed to 1 h for exploration. The ambulatory time and counts, as well as resting time, were generated by ANY-maze software (Stoelting Co., Wood Dale, IL, USA).

#### Novel Object Recognition Test

A novel object recognition test was conducted to assess short-term memory changes in mice, as in our prior reports [28]. The procedure consisted of adaptation, familiarization, and test phases. During acclimation, animals were placed into an open arena (40 × 40 × 32 cm) without any object for 15 min. During familiarization, each mouse was placed into the same chamber including two identical objects (A and B) of similar size and material for 5 min. The accumulated time exploring each object was recorded. During the test phase (after a 1 h retention interval), each mouse was placed into the open field containing two objects (A and C), one of which was identical to the familiarization phase and the other object was novel. The “familiar” and “novel” objects differed dramatically in shape, but not in color, texture, and material. Animals were allowed to freely explore the objects for another 5 min. Object exploration was defined as head orientation, sniffing, and pointing the nose at the object at a distance ≤ 2 cm. The exploration time for the familiar and the novel object was calculated. The discrimination index is considered to be a relative measure of the distinction between the novel object and familiar object (t[novel]- t[familiar]) / (t[novel] + t[familiar]) × 100%.

### Hematoxylin and Eosin (H&E) and Nissl Staining

Mice were anesthetized with pentobarbital sodium (50 mg/kg). The brain tissues were fixed overnight in 4% paraformaldehyde at 4˚C, followed by embedding in paraffin. The blocks were then dehydrated in graded ethanol solutions and transparentized via xylene. The 5-μm-thick coronal sections were obtained using a freezing microtome (Leica Biosystems, Wetzlar, Germany) and stained with hematoxylin and eosin (Solarbio, Beijing, China) or Nissl staining using 0.2% Toluidine Blue solution (Solarbio) for 10 min according to the manufacturer’s instructions. Images were observed and collected by a microscope equipped with a camera. Neuronal profiles with visible nuclei and morphologically intact cells were counted using the Image-pro software, with at least three fields of image per defined region.

### Immunofluorescence Staining

Immunohistochemical protocols were used, as we previously described [29]. After sacrificing the animals, brain tissues were directly fixed with 4% paraformaldehyde overnight, dehydrated with 30% sucrose solution, and embedded in optimal cutting temperature (OCT) compound (Sakura Finetek, Tokyo, Japan). Frozen brain tissues in the hemisphere ipsilateral to the impact site were sliced into coronal slices (40 μm) in a cryostat microtome. Slices were blocked in 0.1 M phosphate-buffered saline (PBS) containing 0.3% Triton X-100, 5% donkey serum, and 2.5% BSA for 2 h at room temperature. Sections were incubated overnight at 4 °C with the following antibodies: anti-CXCL1 (1:100, Boster, Wuhan, Hubei, China; Cat#A00533), anti-CXCL2 (1:100, Bioss, Beijing, China; Cat#bs-1162R), anti-glial fibrillary acidic protein (GFAP) (1:1000, Cell Signal Technology, Danvers, CT, USA; Cat#3670S), anti-neuronal nuclei (NeuN) (1:1,000, CST, Cat#94403S), and anti-ionized calcium binding adapter molecule 1(Iba1) (1:500, Wako, Tokuo, Japan; Cat#019-19741). Next, slides were incubated in Alexa 488-labeled donkey anti-mouse IgG (1:600, Invitrogen, USA; Cat#A-21,202) and Alexa 568-labeled donkey anti-rabbit IgG (1:600, Invitrogen; Cat#A-10,042) for 1 h at room temperature. Slices were then incubated with 4,6-diamidino-2-phenylindole (DAPI) (1:1000, Sigma-Aldrich, USA; Cat#MBD0015) for 30 min and mounted with a coverslip in glycerol. Fluorescence images were obtained using a confocal laser scanning microscope, LSM 980 (Carl Zeiss, Germany). The number of activated microglia and soma in each cell was analyzed using Image J software.

### Western Blot

Brain samples from post-injury cortical tissue were fully lysed with ice-cold radioimmunoprecipitation assay (RIPA) buffer containing 1% protease inhibitor cocktail (Beyotime Biotechnology, Nantong, China). Homogenates were immediately centrifuged at 12,000 g for 10 min at 4˚C. The supernatants were collected, and the protein concentrations were measured using a BCA kit (Keygen Biotech, Nanjing, China). Lysates were denatured for 5 min at 98˚C. Protein samples (30 μg) were separated using 8%/12% sodium dodecyl sulfate-polyacrylamide gel electrophoresis and transferred onto PVDF membranes (Millipore, Billerica, MA, USA). The membranes were blocked with 5% non-fat milk diluted in 0.1% Tween-20 (TBST) for 2 h at room temperature.

Blots were probed overnight at 4˚C with primary antibodies: anti-CXCL1 (1:500, Boster; Cat#A00533), anti-CXCL2 (1:500, Bioss; Cat#bs-1162R), anti-CXCR2 (1:2000, Proteintech, Wuhan, China; Cat#20634-1-AP), anti-ASC (1:1000, CST; Cat#12,242), anti-Caspase-1 p20 (1:500 Santa Cruz Biotechnology, CA, USA; Cat#sc-398,715), anti-NLRP3 (1:1000, Abcam, Cambridge, MA, USA; Cat#ab-263,899), anti-IL-1β (1:1000, CST; Cat#12,242), anti-IL-6 (1:1000, Santa; Cat#sc-32,296), anti-IL-18 (1:1000, Abcam; Cat#ab-207,323) and anti-TNF-α (1:1000, Santa; Cat#sc-52,746). The membranes were incubated with horseradish peroxidase-conjugated goat anti-rabbit IgG (1:5000, ZSGB-Bio, Beijing, China; Cat#ZB-2301) or goat anti-mouse (1:5000, ZSGB; Cat#ZB-2305) secondary antibodies at room temperature for 2 h. β-actin (1:1000, ZSGB-Bio; Cat#TA-09) were used as internal control. Immunoreactive protein bands were developed using super-enhanced chemiluminescence detection reagents on a Bio-Image gel imaging system (Bio-Rad Laboratories, Hercules, CA, USA), and quantified by ImageJ software.

### Statistical Analysis

All results were presented as mean ± standard error of the mean (SEM) and processed by using SPSS 26.0 software (IBM-SPSS Inc, Chicago, IL). Statistical significance was analyzed by Student’s *t*-test and two-way analysis of variance (ANOVA) with Tukey’s *post hoc* test. *p* < 0.05 was considered as the significance threshold.

## Results

### SB332235 Mitigates TBI-Induced Neurological Deficits

Representative images of the whole brain in mice following TBI depicted obvious damage compared to Sham-operated animals (Sham, Fig. 1b). Meanwhile, TBI mice exhibited severe and noticeable neurological dysfunction with an average mNSS score (8.88 ± 0.32 points, *n* = 16, *p* < 0.001, Fig. 1c), whereas Sham mice were healthy without any neurological dysfunction and mNSS score of 0.31 ± 0.12 points, *n* = 16. This validates the successfully generating the TBI mice model using the controlled cortical impact method.

To evaluate the neuroprotective effect of SB332235 on neurological impairments induced by TBI, 1 mg/kg SB332235 was administrated (i.p.) at 30 min post-TBI, followed by additional doses at 6, 24, and 30 h (Fig. 1a). mNSS was evaluated on day 3 post-TBI. Two-way ANOVA analysis revealed the TBI × SB332235 interaction effects on mNSS scores [F _(1, 60)_ = 308.77, *p* < 0.001]. Post-hoc analysis demonstrated TBI mice treated with SB332235 (TBI + SB) had significantly smaller mNSS scores (1.44 ± 0.22 points, *n* = 16) relative to that of the TBI group (*p* < 0.001, Fig. 1c), indicating neuroprotective effects of SB332235 which mitigated TBI induced neurological deficits.

### SB332235 Ameliorates TBI-Induced Behavioral Deficits

**Fig. 2:**
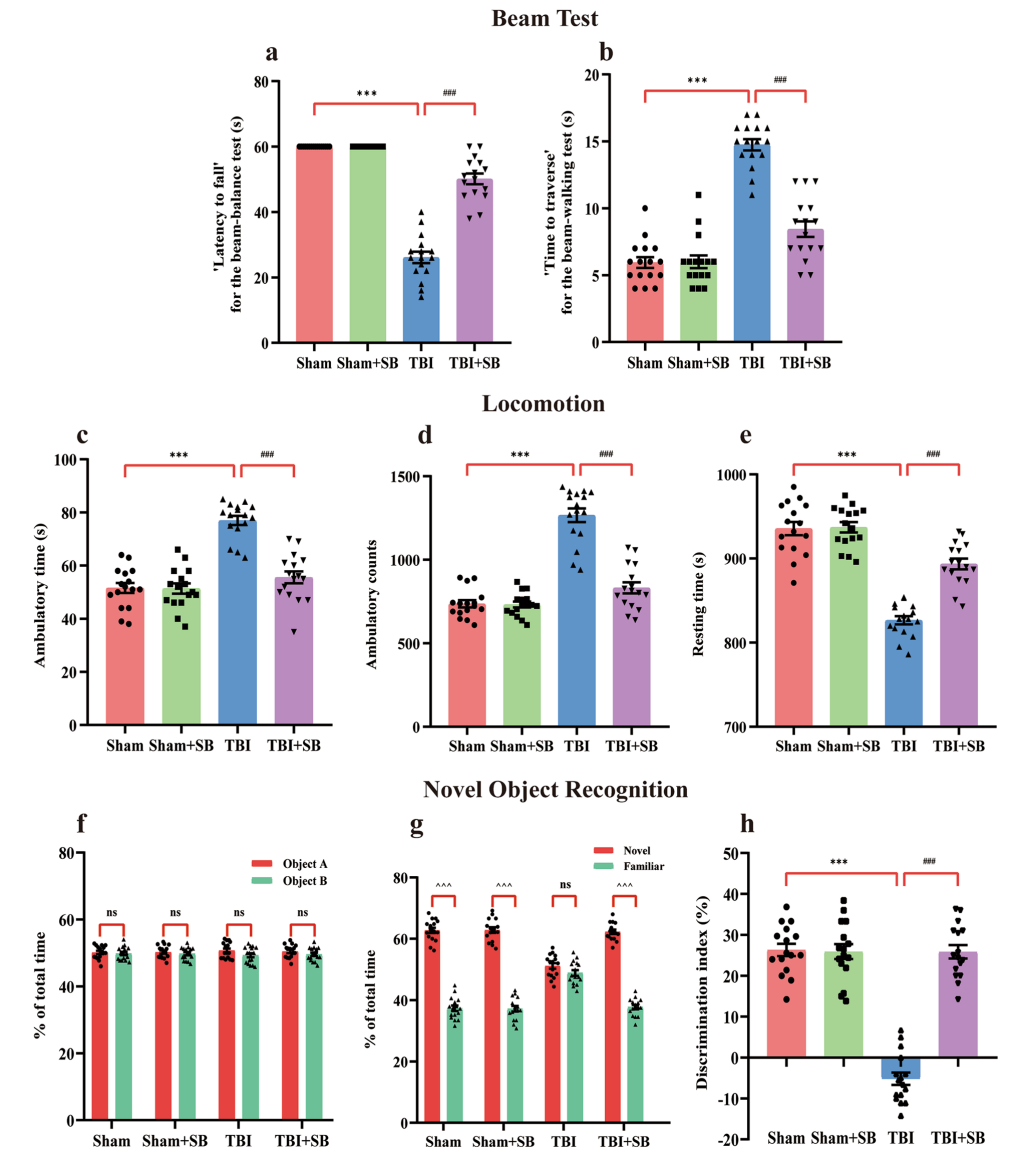
SB332235 ameliorates TBI-induced motor dysfunction and recognition impairment. (**a**) Bar graphs of balance and traversal time from the beam-balance test and (**b**) beam-walk test. (**c-e**) Open field test was performed to quantify the ambulatory time (**c**) and counts (**d**), and resting time (**e**). (**f-h**) Bar graphs of the percentage of total time spent exploring similar objects during familiarization (**f**), the percentage of total time spent exploring novel and familiar objects (**g**), and the discrimination index (**h**) during the test phase of the novel object recognition test. Data are presented as mean ± SEM (*n* = 16 mice per group). Two-way ANOVA with Tukey’s *post hoc* test: ^***^p < 0.001 vs. Sham group; ^###^*p* < 0.001 vs. TBI group; ^^^^^*p* < 0.001 for novel object vs. familiar object, “ns” indicates no statistical significance

We next investigated the effects of SB332235 on behavioral phenotypes of the TBI mouse model, focusing on motor and memory performance, which are hallmarks of TBI in humans [27, 30]. Beam-balance and beam-walk tests were used to evaluate gross and fine motor functions. Mice with TBI showed less latency to fall in the beam balance test [Two-way ANOVA, F _(1, 60)_ = 99.35, *p* < 0.001; Sham = 60 ± 0 s, *n* = 16; TBI = 26.13 ± 1.75 s, *n* = 16; *post hoc*, *p* < 0.001] but longer time to traverse the beam in the beam-walk test [Two-way ANOVA, F _(1, 60)_ = 138.85, *p* < 0.001; Sham = 5.94 ± 0.40 s, *n* = 16; TBI = 14.75 ± 0.42 s, *n* = 16; *post hoc*, *p* < 0.001] when compared to those of Sham group (Fig. 2a and b). These data suggest fine motor dysfunction caused by TBI. While with SB332235 treatment, mice in TBI + SB group had improved fine motor performance with longer latency to fall in beam-balance test (TBI + SB = 50.13 ± 1.65 s, *n* = 16, *post hoc*, *p* < 0.001) and decreased time to traverse the beam in the beam-walking test (TBI + SB = 8.44 ± 0.58 s, *n* = 16, *post hoc*, *p* < 0.001) compared to those of TBI group (Fig. 2a and b).

The locomotor behavior was analyzed using the open-field test. Compared to the Sham group, mice with TBI exhibited increased ambulatory time [Two-way ANOVA, F _(1, 60)_ = 29.52, *p* < 0.001; Sham = 51.56 ± 1.91 s, *n* = 16; TBI = 77 ± 1.72 s, *n* = 16; *post hoc*, *p* < 0.001], ambulatory count [Two-way ANOVA, F _(1, 60)_ = 52.39, *p* < 0.001; Sham = 736 ± 21.47, *n* = 16; TBI = 1262 ± 40.72, *n* = 16; *post hoc*, *p* < 0.001], and decreased resting time [Two-way ANOVA, F _(1, 60)_ = 25.98, *p* < 0.001; Sham = 935.6 ± 7.91 s, *n* = 16; TBI = 826.6 ± 4.92 s, *n* = 16; *post hoc*, *p* < 0.001] (Fig. 2c and e), indicating TBI-induced hyperactivity in the acute phase. Nevertheless, treatment with SB332235 improved locomotor functioning, with decreased ambulatory time (TBI + SB = 55.56 ± 2.25 s, *n* = 16, *post hoc*, *p* < 0.001), ambulatory count (TBI + SB = 831.1 ± 33.24, *n* = 16, *post hoc*, *p* < 0.001), and increased resting time (TBI + SB = 893.5 ± 6.39 s, *n* = 16, *post hoc*, *p* < 0.001) when compared to those of the TBI group (Fig. 2c and e).

To test the object recognition memory, we analyzed the time mice took to discriminate between a new and familiar object. In the familiarization phase, the exploration time of the two identical objects was analogous for all groups [Two-way ANOVA, F _(1, 60)_ = 0.10, *p* > 0.05, Fig. 2f]. However, during the retention session, mice treated with TBI exhibited a decreased percentage of exploration time spent with the novel object [F _(1, 60)_ = 40.58; Sham = 62.74 ± 0.87%, *n* = 16; TBI = 51.14 ± 0.93%, *n* = 16; *post hoc*, *p* < 0.001] and a lower discrimination index [Two-way ANOVA, F _(1, 60)_ = 94.33, *p* < 0.001; Sham = 26.31 ± 1.5%, *n* = 16; TBI = -5.18 ± 1.52%, *n* = 16; *post hoc*, *p* < 0.001] compared to those of Sham group (Fig. 2g and h). However, brain-injured mice treated with SB332235 significantly increased the amount of time directed to the exploration of the novel object (TBI + SB = 62.28 ± 0.7%, *n* = 16, *post hoc*, *p* < 0.001, Fig. 2g) and augment the discrimination ratio [TBI + SB = 25.86 ± 1.63%, *n* = 16, *post hoc*, *p* < 0.001, Fig. 2h] when compared to the TBI group. Altogether, these findings suggest that SB332235 administration effectively rescues TBI-induced behavioral deficits, including motor and memory impairments.

### SB332235 Reduces TBI-Induced Brain Damage

**Fig. 3:**
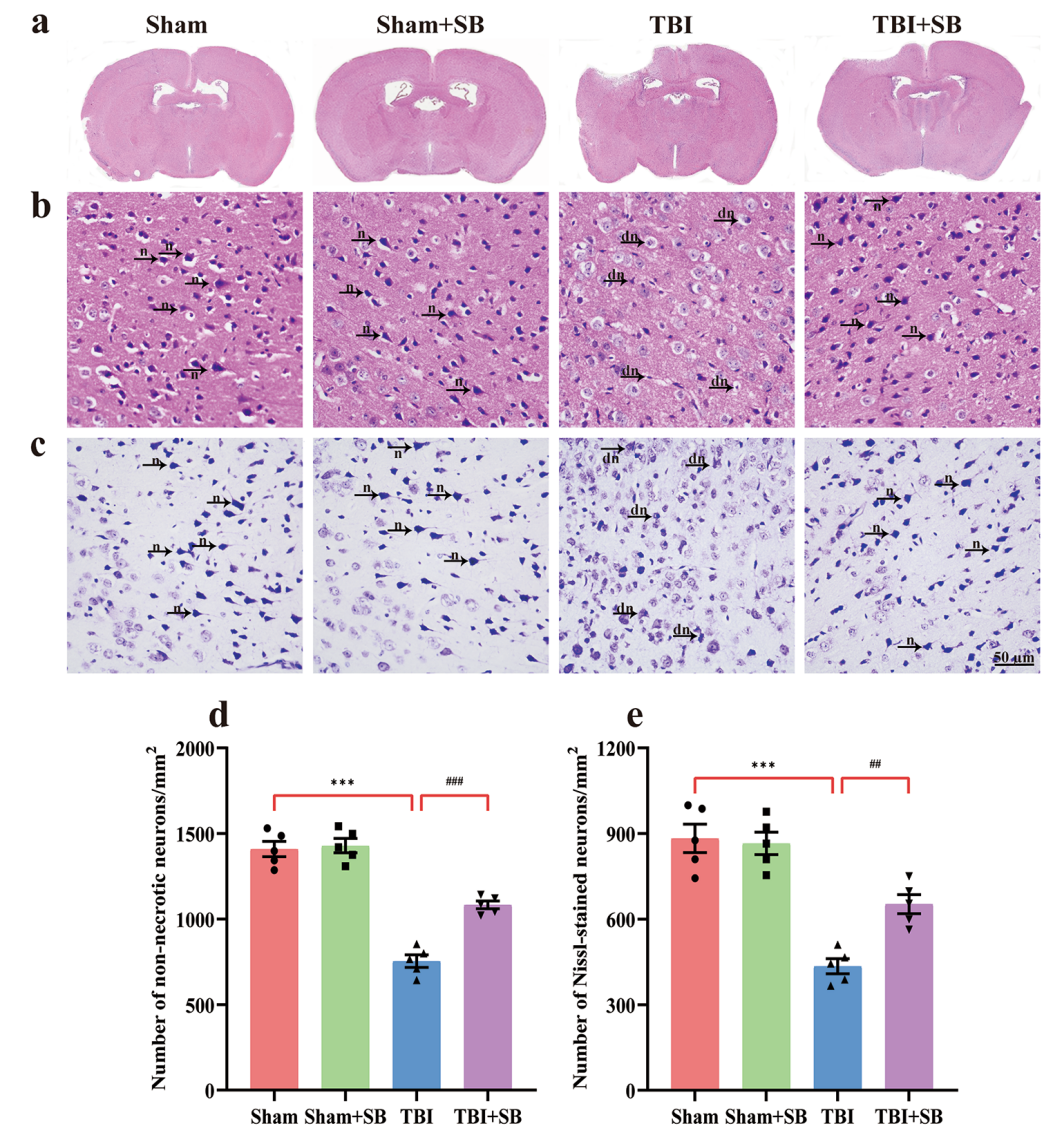
Protective effects of SB332235 on TBI-induced pathological changes. (**a**) Representative scanning images of HE-stained brain sections surrounding the cortical contusion site. The brain tissues of the Sham group were intact, whereas the TBI group displays visible cortical tissue damage. However, cortical damage in the TBI + SB group is significantly reduced. (**b**) Representative photomicrographs of HE-stained neurons. Black arrows indicate n-normal pyramidal neurons and intact parenchyma, while dn denotes neuronal vacuolation, shrinkage, and degeneration. (**c**) The Nissl staining images of cortical neurons. Black arrows indicate n-normal Nissl substance staining, while dn denotes neuronal swelling, edema, shrinkage, and irregular shape. (**d**) Counts of non-necrotic neurons from H&E staining. (**e**) Cell counts by Nissl staining. Data are presented as mean ± SEM (*n* = 15 slices from 5 mice per group). Two-way ANOVA with Tukey’s *post hoc* test: ^***^p < 0.001 vs. Sham group; ^##^*p* < 0.01, ^###^*p* < 0.001 vs. TBI group

To identify the pathological changes, we carried out H&E staining to detect the morphological characteristics, and Nissl staining for neuronal viability. As shown in Fig. 3a and b, peri-lesional regions of TBI mice exhibited significant pathological changes relative to the Sham group. The brain tissues showed uneven breakage and the cell membranes of neurons were less intact, with signs of vacuolization, granulovacuolar neuronal degeneration, parenchymal loss, neuronal wither, and neuronal edema induced by TBI. While counting the non-necrotic neurons, decreased number of undamaged neurons were found in the TBI animals [Two-way ANOVA, F _(1, 16)_ = 21.67, *p* < 0.001; 1409 ± 45.04/mm^2^, *n* = 5, TBI = 754.2 ± 36.33/mm^2^, *n* = 5; *post hoc*, *p* < 0.001, Fig. 3d]. Animals treated with SB332235 showed relatively smaller damaged brain regions (Fig. 3a), accompanied by increased non-necrotic neurons (TBI + SB = 1083 ± 22.92/mm^2^, *n* = 5, *post hoc*, *p* < 0.001, Fig. 3d) when compared to those of TBI mice.

Similarly, Nissl staining showed abnormal neuronal morphology in slices from TBI mice, with irregular cell bodies, shrinkage, and hyperchromatic nuclei (Fig. 3c). Cell counting found fewer Nissl-stained neurons in slices from TBI mice [Two-way ANOVA, F _(1, 16)_ = 7.00, *p* < 0.05; Sham = 883 ± 49.62/mm^2^, *n* = 5, TBI = 434.8 ± 26.11/mm^2^, *n* = 5; *post hoc*, *p* < 0.001, Fig. 3e]. However, slices from TBI + SB332235 mice showed extensive blue granular Nissl bodies, indicating normal morphology (Fig. 3c) and an increased number of Nissl-stained neurons compared to TBI mice (TBI + SB = 652.6 ± 33.44/mm^2^, *n* = 5, *post hoc*, *p* < 0.01, Fig. 3e).

In summary, these results indicate that SB332235 treatment can prevent neuronal damage in the perilesional cortex after brain trauma, as evidenced by improvements in neuronal morphology and viability.

### Localization of CXCL1 and CXCL2 After TBI

**Fig. 4:**
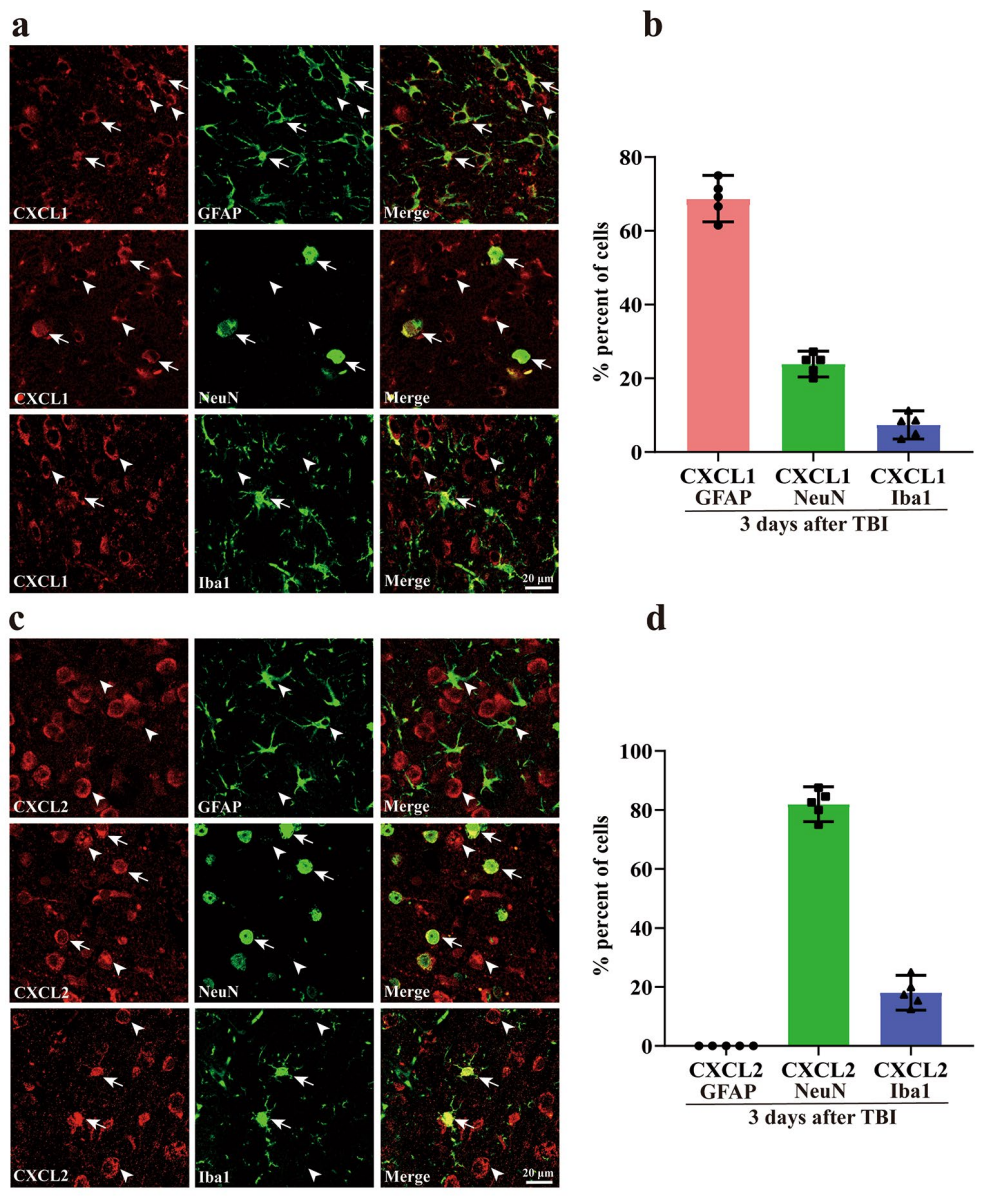
The localization of CXCL1 and CXCL2 in the ipsilateral cortex after TBI. (**a**) Double immunofluorescence staining of CXCL1 (red) with astrocyte (GFAP), neuron (NeuN), and microglia/macrophages (Iba1) (green) markers in the lesioned cortex. Colocalization (yellow, indicated with arrows) can be seen in the overlay; arrowheads show lack of colocalization. (**b**) Bar graph of co-labeling rate of CXCL1 with different cell types. (**c-d**) Similar to a-b but with CXCL2 staining. Data are resented as mean ± SEM (*n* = 5 per group)

Several high-affinity endogenous ligands for CXCR2 have been identified such as the chemokine CXCL1 and CXCL2, which participate in the inflammatory response caused by TBI [21, 31, 32]. To identify the cellular localization of CXCL1 and CXCL2 in the mouse cortex at 3 days after TBI, we employed immunofluorescence double staining of CXCL1 and CXCL2 with the astrocyte marker GFAP, the neuronal marker NeuN, and the microglial marker Iba1, respectively. As shown in Figs. 4a and b, 68.74 ± 2.27% (*n* = 5) of CXCL1 was co-labeled with GFAP, 23.88 ± 1.25% (*n* = 5) with NeuN, and 7.38 ± 1.38% (*n* = 5) with Iba1. While, 81.94 ± 2.13% (*n* = 5) of CXCL2 was co-labeled with the NeuN and 18.06 ± 2.13% (*n* = 5) with the Iba1, but without co-labeled with GFAP (Fig. 4c and d). These results indicate that CXCL1 predominantly expresses in astrocytes but CXCL2 in neurons in the injured cortex following TBI.

Additionally, we found that CXCR2-containing cells colocalized with the neuronal marker NeuN in the cortex of Sham groups. The double immunofluorescence staining of CXCR2 with the microglia marker Iba1 was detected at 3 days post-TBI. We observed that CXCR2 was mainly expressed in activated microglia in the peri-contusional cortex of mice (Supplementary Fig. 1). These results are consistent with previous research [33, 34]. Our data could not exclude the possibility that CXCR2 was also expressed in neurons following TBI.

### SB332235 Suppresses CXCL1, CXCR2 Expression and NLRP3 Inflammasome Caused by TBI

**Fig. 5:**
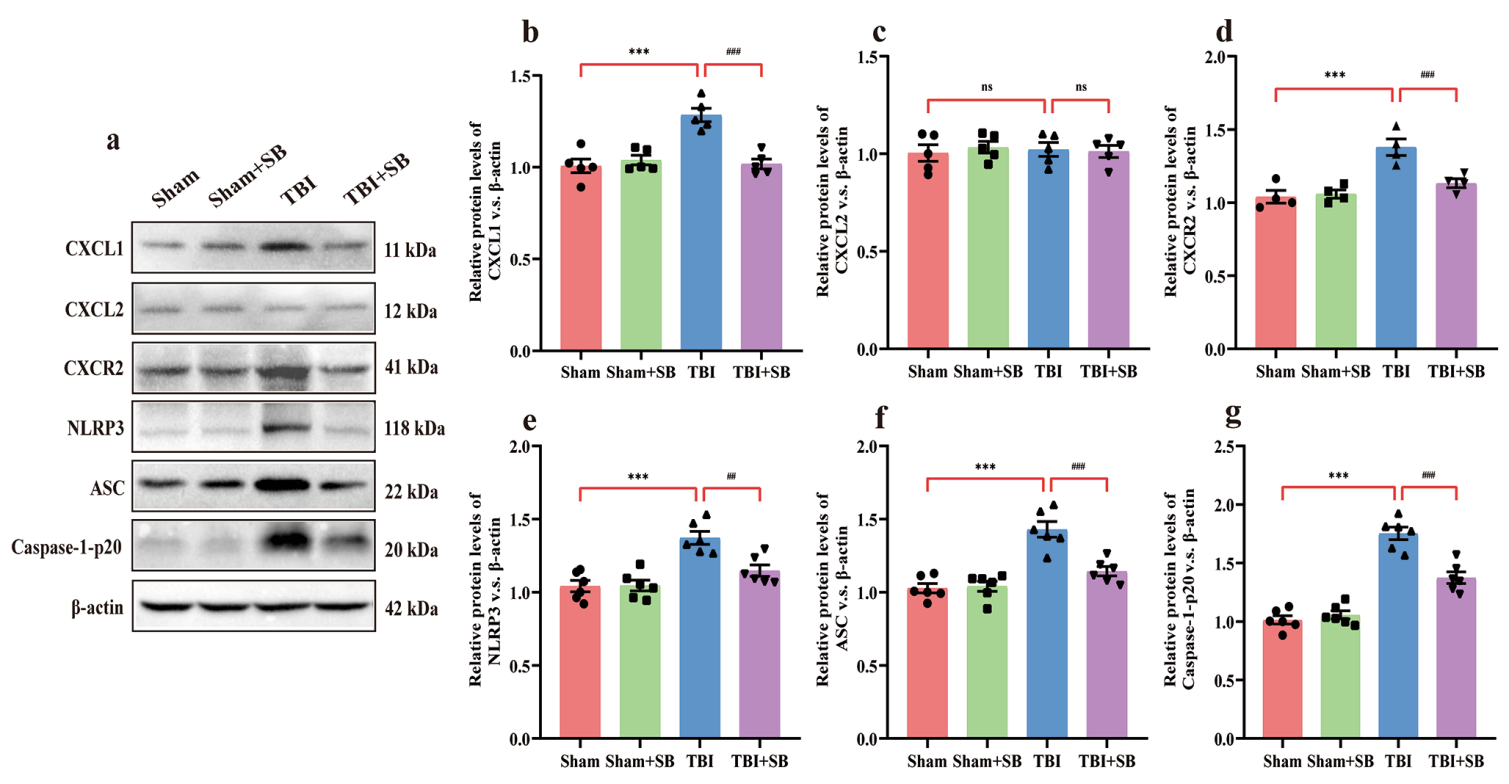
SB332235 downregulates CXCL1, CXCR2 and NLRP3 inflammasome surrounding the injured brain. (**a**) Representative immunoblots of WB. (**b-g**) Quantification of CXCL1, CXCL2, CXCR2, NLRP3, ASC and caspase-1 p20. Data are presented as mean ± SEM (*n* = 5 brains per group for CXCL1 and CXCL2; *n* = 4 per group for CXCR2; *n* = 6 per group for NLRP3, ASC and caspase-1 p20). Two-way ANOVA with Tukey’s *post hoc* test: ^***^p < 0.001 vs. Sham group; ^##^*p* < 0.01, ^###^*p* < 0.001 vs. TBI group. “ns” indicating no statistical significance

We measured whether CXCL1 and CXCL2 levels in TBI-treated mice were reversed by SB332235 treatment. As illustrated in Fig. 5a and b, significant upregulation of CXCL1 was found in the ipsilateral contused cortex in TBI mice compared to the Sham-operated animals [Two-way ANOVA, F _(1,16)_ = 16.92, *p* < 0.001; Sham = 1.01 ± 0.04, *n* = 5; TBI = 1.29 ± 0.04, *n* = 5; *post hoc*, *p* < 0.001]. However, SB332235 administration dramatically mitigated TBI-induced CXCL1 over expression [TBI + SB = 1.02 ± 0.03, *n* = 5, *post hoc*, *p* < 0.001], when compared to TBI animals. Notably, no significant changes of CXCL2 were detected between different groups [Two-way ANOVA, F _(1, 60)_ = 0.14, *p* > 0.05, *n* = 5; Fig. 5a and c].

The chemokine receptor CXCR2 is stimulated by the chemokine CXCL1 [18]. Compelling evidence suggests that TBI patients exhibit CXCR2 overexpression on monocytes, and CXCR2 activation in microglia induces NLRP3 inflammasome activation [18, 20]. Mice with TBI showed an increase in CXCR2 levels [Two-way ANOVA, F _(1,12)_ = 10.27, *p* < 0.01; Sham = 1.04 ± 0.04, *n* = 4; TBI = 1.38 ± 0.06, *n* = 4; *post hoc*, *p* < 0.001] in the mouse cortex around an injury site, when compared with Sham controls (Fig. 5a and d). Whereas administration of SB332235, a selected CXCR2 antagonist, effectively ameliorated such augmentation of CXCR2 protein expression (TBI + SB = 1.13 ± 0.03, *n* = 4, *post hoc*, *p* < 0.001) relative to the brain-injured mice (Fig. 5a and d).

NLRP3 inflammasome is reported to play an essential role in the inflammatory response following TBI. This inflammasome is composed of an adaptor protein called protein-apoptosis-associated speck-like protein, encompassing a CARD (ASC) and caspase-1 [14]. In the present study, we investigated the impact of SB332235 on the activation of the NLRP3 inflammasome in TBI. Specifically, we examined protein expression of NLRP3, ASC, and cleaved activated Caspase-1 (Caspase-1 p20) in the lesioned boundary post-TBI using Western blotting.

Mice exposed to TBI demonstrated a substantial elevation in protein expression of NLRP3 [Two-way ANOVA, F _(1,20)_ = 8.29, *p* < 0.01; Sham = 1.04 ± 0.04, *n* = 6; TBI = 1.37 ± 0.04, *n* = 6; *post hoc*, *p* < 0.001], ASC [Two-way ANOVA, F _(1,20)_ = 14.56, *p* < 0.001; Sham = 1.03 ± 0.03, *n* = 6; TBI = 1.43 ± 0.05, *n* = 6; *post hoc*, *p* < 0.001], and Caspase-1 p20 [Two-way ANOVA, F _(1,20)_ = 22.88, *p* < 0.001; Sham = 1.01 ± 0.04, *n* = 6; TBI = 1.75 ± 0.05, *n* = 6; *post hoc*, *p* < 0.001] when compared to Sham-operated controls (Fig. 5a and 5e-g). However, SB332235 treatment significantly reduced the protein expression of NLRP3 (TBI + SB = 1.15 ± 0.04, *n* = 6, *post hoc*, *p* < 0.01), ASC (TBI + SB = 1.14 ± 0.03, *n* = 6, *post hoc*, *p* < 0.001) and Caspase-1 p20 (TBI + SB = 1.37 ± 0.05, *n* = 6, *post hoc*, *p* < 0.001) compared to the TBI group (Figs. 5a and 5e-g). Overall, our findings indicate that NLRP3 inflammasome is activated following TBI and SB332235 can effectively inhibit this effect.

### SB332235 Reduces Inflammatory Factors in TBI

**Fig. 6:**
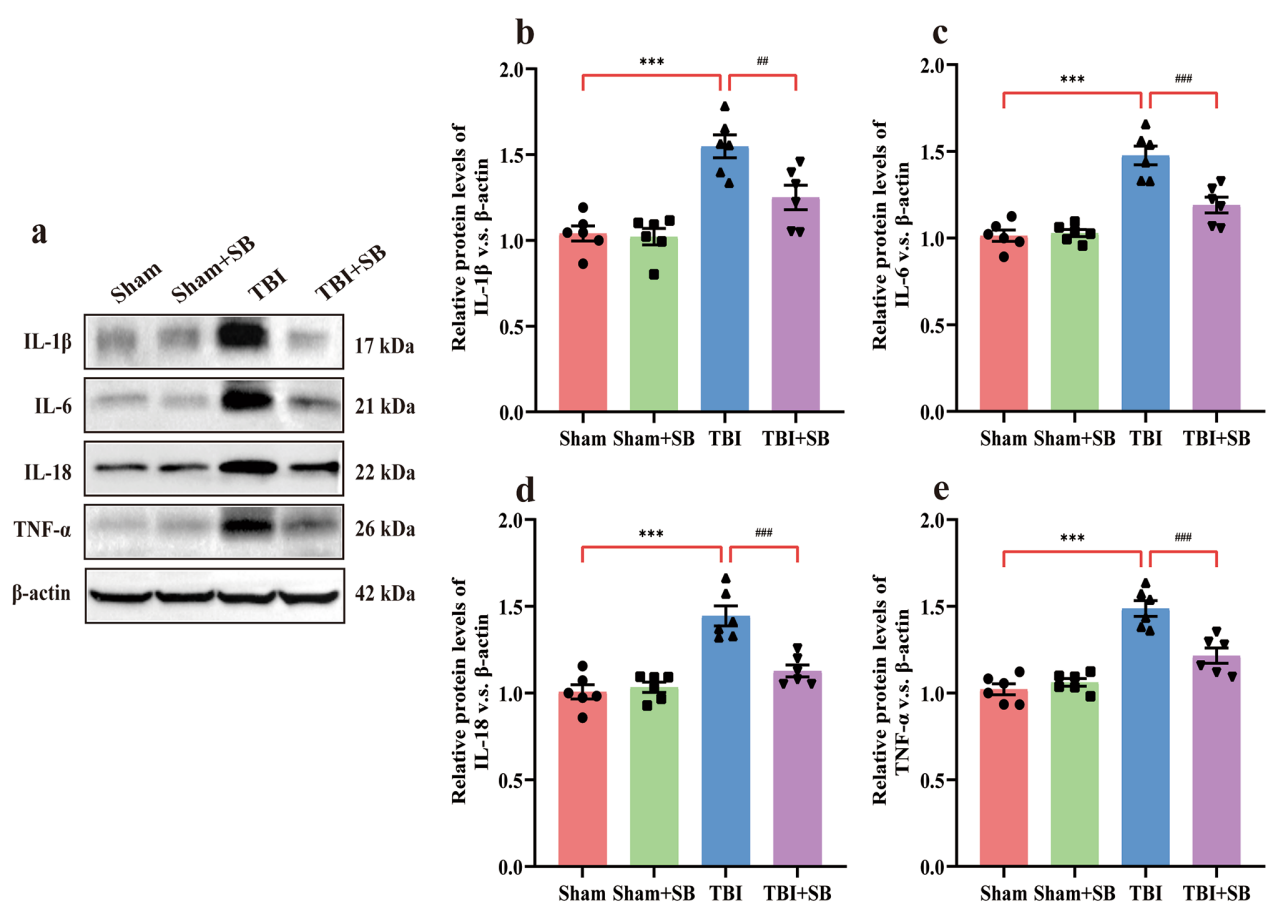
Effects of SB332235 on TBI-induced inflammatory factor expression. (**a**) Representative immunoblots of WB. (**b-e**) Quantification of IL-1β, IL-6, IL-18, and TNF-α in the lesioned cortex. Data are presented as mean ± SEM (*n* = 6 brains per group). Two-way ANOVA with Tukey’s *post hoc* test: ^***^p < 0.001 vs. Sham group; ^##^*p* < 0.01, ^###^*p* < 0.001 vs. TBI group

It is well established that the production of proinflammatory factors is associated with the activation of NLRP3 inflammasome proteins in TBI [14]. To investigate whether the repression of NLRP3 by SB332235 could decrease the levels of pro-inflammatory cytokines, we tested the IL-1β, IL-6, IL-18, and TNF-α levels in the peri-lesional cortex. Mice with TBI exhibited upregulated protein expression of IL-1β [Two-way ANOVA, F _(1, 20)_ = 5.67, *p* < 0.05; Sham = 1.04 ± 0.04, *n* = 6; TBI = 1.55 ± 0.07, *n* = 6; *post hoc*, *p* < 0.001], IL-6 [Two-way ANOVA, F _(1, 20)_ = 14.09, *p* < 0.001; Sham = 1.01 ± 0.03, *n* = 6, TBI = 1.48 ± 0.05, *n* = 6; *post hoc*, *p* < 0.001], IL-18 [Two-way ANOVA, F _(1, 20)_ = 16.76, *p* < 0.001; Sham = 1.01 ± 0.04, *n* = 6; TBI = 1.45 ± 0.06, *n* = 6; *post hoc*, *p* < 0.001] and TNF-α [Two-way ANOVA, F _(1, 20)_ = 15.17, *p* < 0.001; Sham = 1.02 ± 0.03, *n* = 6; TBI = 1.49 ± 0.05, *n* = 6; *post hoc*, *p* < 0.001] compared to Sham-operated mice (Fig. 6a and e). Whereas SB332235 treatment remarkably attenuates the TBI-induced elevations of these inflammatory cytokines, including IL-1β (TBI + SB = 1.25 ± 0.07, *n* = 6, *post hoc*, *p* < 0.01), IL-6 (TBI + SB = 1.19 ± 0.05, *n* = 6, *post hoc*, *p* < 0.001), IL-18 (TBI + SB = 1.13 ± 0.03, *n* = 6, *post hoc*, *p* < 0.001) and TNF-α (TBI + SB = 1.22 ± 0.04, *n* = 6, *post hoc*, *p* < 0.001; Fig. 6a and e). These findings suggest that SB332235 could attenuate neuroinflammation caused by TBI.

### SB332235 Mitigates TBI-Induced Microglial Activation

**Fig. 7:**
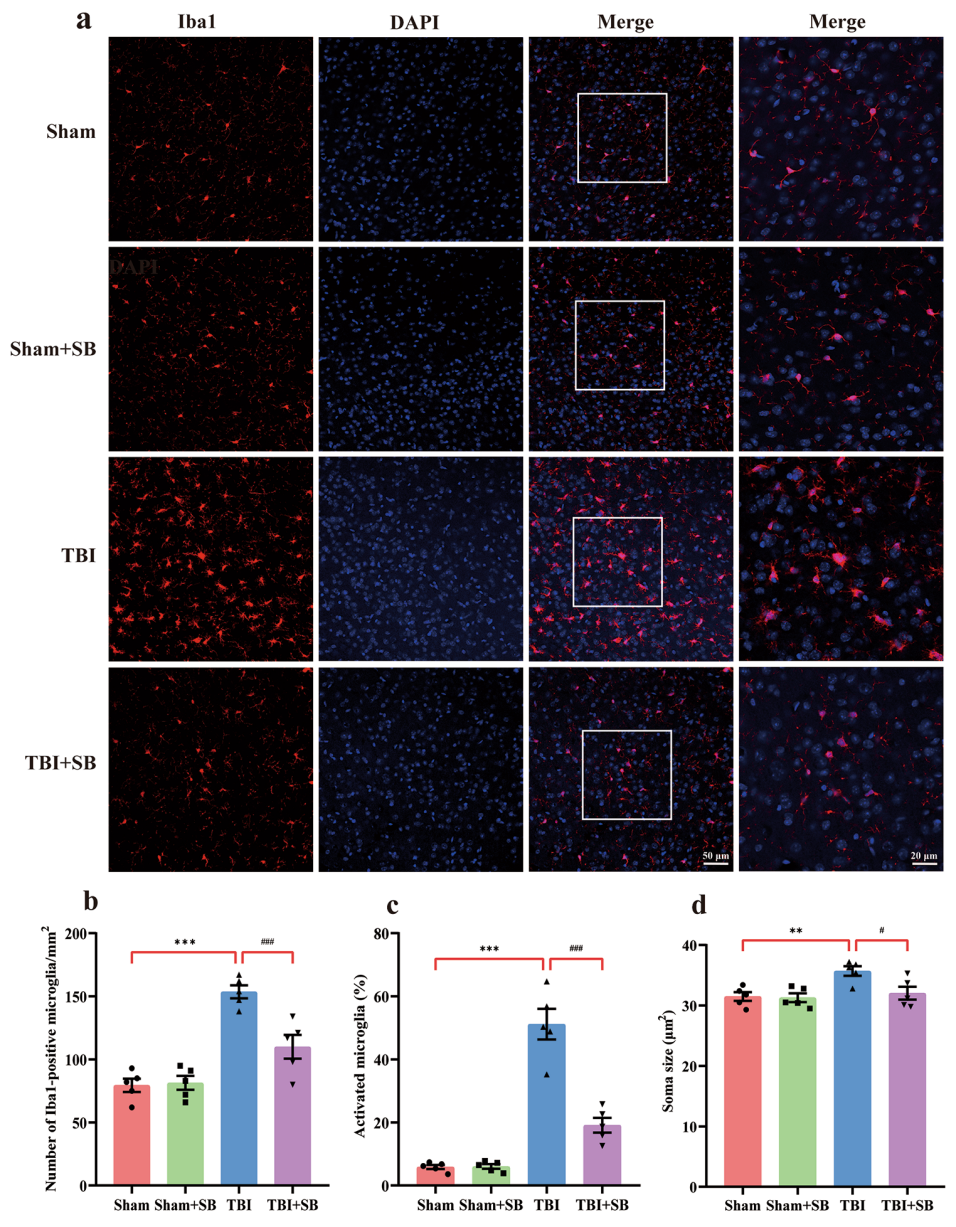
SB332235 mitigates activated microglia post-injury. (**a**) Immunofluorescence staining of microglial marker Iba1 (red) around the injury site. DAPI was used to counterstain cell nuclei (blue). High-magnitude images exhibited de-ramified microglia. (**b**) Quantification of total microglia number. (**c**) The percentage of activated microglia. (**d**) Iba1-stained area. Data are presented as mean ± SEM (*n* = 15 slices from 5 mice per group). Two-way ANOVA with Tukey’s *post hoc* test: ^**^p < 0.01, ^***^p < 0.001 vs. Sham group; ^#^*p* < 0.05, ^###^*p* < 0.001 vs. TBI group

Microglial activation is a well-known hallmark of TBI [11]. We next evaluated the potential of SB332235 to alleviate the neuroinflammatory response following TBI. Immunofluorescence labeling against Iba1 was used to detect microglia/macrophages [35]. As shown in Fig. 7a, microglial activation occurred in the peri-contusional tissue of injured animals, which displayed smaller cell bodies and a fine appearance. Activated microglia (e.g., de-ramified morphology) displayed a hypertrophic and ramification disappearance in injured animals. Treatment with SB332235 inhibited microglial aggregation induced by TBI.

Mice exposed to TBI showed a significant increase in the total number of microglia [Two-way ANOVA, F _(1, 16)_ = 11.96, *p* < 0.01; Sham = 79.4 ± 5.22/mm^2^, *n* = 5; TBI = 153.6 ± 5.19/mm^2^, *n* = 5, *post hoc*, *p* < 0.001], the percentage of activated microglia [Two-way ANOVA, F _(1, 16)_ = 34.56, *p* < 0.001; Sham = 5.88 ± 0.66%, *n* = 5; TBI = 51.16 ± 4.88%, *n* = 5; *post hoc*, *p* < 0.001], and Iba1-stained area [Two-way ANOVA, F _(1, 16)_ = 4.32, *p* < 0.05; Sham = 31.48 ± 0.72/μm^2^, *n* = 5; TBI = 35.7 ± 0.78 /μm^2^, *n* = 5; *post hoc*, *p* < 0.01] compared to those of Sham animals (Fig. 7b and d). Of particular interest, SB332235 administration significantly prevented these changes with the decreased total number of microglia (TBI + SB = 110 ± 9.46/mm^2^, *n* = 5, *post hoc*, *p* < 0.001), the percentage of activated microglia (TBI + SB = 19.12 ± 2.34%, *n* = 5, *post hoc*, *p* < 0.001), and Iba1-stained area (TBI + SB = 32.02 ± 1.07/μm^2^, *n* = 5, *post hoc*, *p* < 0.05) when compared to the TBI group (Fig. 7b and d). Taken together, our findings suggest that SB332235 treatment inhibits microglial activation due to TBI-induced neuroinflammation.

## Discussion

It is widely accepted that reducing neuroinflammation is a favorable approach for attenuating secondary brain injuries and improving neurological outcomes following TBI [15, 36, 37]. Accumulating evidence indicates CXCR2 antagonist, SB332235 possesses anti-inflammatory properties in central nervous system disorders [19, 20, 38]. The current study aimed to investigate whether the administration of SB332235 could attenuate TBI-induced neurological deficits, and motor and cognitive impairments in mice. Our results showed that SB332235 administration improved functional outcomes, which was associated with reduced CXCL1 and CXCR2 levels, and suppressed the activation of NLRP3 inflammasome surrounding the traumatic lesions. This was accompanied by blocking downstream pro-inflammatory cytokine production. In addition, SB332235 inhibited excessive microglial activation. Our findings elucidated that SB332235 exerts neuroprotective effects in a mouse model of TBI, at least in part, by suppressing the NLRP3 inflammasome signaling cascade. These data indicate that SB332235 may be a promising pharmacotherapeutic target for promoting functional recovery after brain injury.

It is well documented that TBI leads to extensive damage of neuronal associated with behavioral disturbances [14, 39]. In this study, SB332235 was capable of ameliorating the neuronal damage and improved subsequent motor and cognitive deficits at 3 days post-injury. In parallel with previous studies [27, 40–42], our data showed that TBI caused pronouncedly higher mNSS score, reduction of time to maintain balance in beam-balance, enhancement in ambulatory time and a concomitant diminution in resting time in an open field, and reduced discrimination index in novel object recognition task in animals, suggesting that TBI can trigger neurological deficiency, motor, and cognitive dysfunction. Of particular noteworthy, treatment with SB332235 potently reversed such deficits as shown by the lower mNSS scores, elevation of time to maintain balance, decrease in ambulatory time and increase in resting time, and enhancement of discrimination index in TBI-exposed animals, indicative of a beneficial effect of SB332235 on the motor and memory impairments. Moreover, administration of SB332235 increased neuronal viability in the peri-contusion area impaired by TBI insult, as manifested via HE and Nissl staining. Our results are in accordance with those of recent findings [8, 43]. In practice, increasing evidence has demonstrated that SB332235 reduced neuronal degeneration and apoptosis in animals [21, 44]. Accordingly, the present work further proposed that CXCR2 antagonist SB332235 was able to rescue TBI-induced motor and cognitive defects via alleviating neuronal damage.

As a G protein-coupled receptor, CXCR2 is activated by CXC chemokines including murine CXCL1. Activation of CXCR2 by its ligand CXCL1 has been detected in TBI [31], and CXCR2 inhibition improved the neurological function of animal models of TBI [21]. Currently, we show that the chemokine CXCL1 and its receptor CXCR2 are prominently upregulated in the ipsilateral cortex surrounding the lesion in mice. In keeping with our views, Huang et al. [25] identified pronounced elevation of CXCL1 and CXCR2 expression in the injured cortex at 3 days following TBI. Furthermore, we confirmed that CXCL1 is predominantly expressed in astrocytes in the cortical damaged area, this is similar to what have been demonstrated in the rat cortex [21, 25]. Under normal conditions, CXCR2 is mainly expressed in neurons [33]. Also, immunofluorescence staining demonstrated that CXCR2 was highly expressed in activated microglia of chronic ischemia-induced white matter injury [34]. Of particular noteworthy, we discovered that administration of competitive CXCR2 antagonist SB332235 resulted in the substantial decrease in CXCL1 and CXCR2 levels at 3 days post TBI. In essence, application of SB332235 to Aβ_1–42_ peptide-injected animals inhibited CXCR2 expression in molecular layer region of dentate gyrus [44]. These observations provide strong evidence that SB332235 effectively blocked CXCL1-CXCR2 mediated the interaction between activated astrocytes and microglia thus successfully improved motor and cognition functions upon trauma.

Previous studies have revealed interaction between CXCL1 and CXCR2 can modulate the activation of the NLRP3 inflammasome [18]. Inflammatory responses mediated by the NLRP3 inflammasome contribute to motor and cognitive impairments following TBI [12, 14, 45]. Research suggests that inhibiting the activation of NLRP3 inflammasome alleviates neuroinflammation histopathological deficits, as well as improve functional outcome in TBI animal models [37, 46]. After TBI, NLRP3 acts as a sensor molecule that undergoes self-oligomerization. Oligomerized NLRP3 recruits ASC and results in the aggregation of ASC into a macromolecular focus. The assembled ASC then recruits procaspase-1 via CARD-CARD interactions to form the NLRP3 inflammasome. Subsequently, it causes pro-caspase-1 self-cleavage and activation, which facilitates the release of pro-inflammatory cytokines, such as IL-1β and IL-18 [25]. IL-1β production is coordinated with that of TNF-α, which exacerbates secondary brain injury [47]. Consistent with previous findings [48–50], we observed the remarkable increase in protein levels of NLRP3 inflammasome components, including NLRP3, ASC, and cleaved caspase-1, as well as increased production of pro-inflammatory factors (such as IL-1β, IL-6, IL-18, and TNF-α) in the lesioned cortical region. These elevations were blocked by the administration of SB332235 which also restores motor and cognitive deficits, indicating a notable protective effect of SB332235 against NLRP3 inflammasome activation after TBI.

NLPR3 inflammasome is predominantly expressed in microglia, which is thought to significantly contribute to brain tissue damage and neurological impairments after TBI [16]. Du et al. [9] demonstrated that activated microglia is associated with sustained augmentation in the expression of pro-inflammatory cytokines such as IL-1β and TNF-α. Another study demonstrated that SB332235 inhibits CXCR2 activity in activated microglia, thereby reducing inflammatory responses and promoting recovery following TBI [44]. In this study, we further investigated the effects of SB332235 on TBI-induced inflammatory responses. Microglia exhibit a notably ramified morphology under basal or “resting” conditions, and de-ramification is considered an enhanced classical activation in inflammatory conditions [51, 52]. Consistent with previous findings [8, 53, 54], we discovered that the number of Iba1-containing cells with activated microglia was strikingly augmented on day 3, along with morphological changes in the injured site. Microglia became incrementally de-ramified in TBI-exposed mice, as characterized by enlarged cell bodies and shorter and thicker processes. Our results revealed that SB332235 treatment inactivated microglia and impeded the expression levels of pro-inflammatory cytokines caused by TBI. Future studies focusing on genetic profiling of de-ramified microglia will be an important challenge in understanding the anti-inflammatory effect of SB332235 after brain injury.

In summary, our data provides evidence for the potential therapeutic use of SB332235 in the treatment of TBI. Further research aimed at translating these findings into clinical applications would be highly advantageous, especially with regard to the function of SB33223 in inhibiting the NLRP3 inflammasome pathway in TBI.

### Electronic Supplementary Material

Below is the link to the electronic supplementary material.

Supplementary Material 1

## Data Availability

All data supporting the conclusions of this article are available from the corresponding author upon reasonable request.
