# Supplementary material for: Neuroprotective Effects of CXCR2 Antagonist SB332235 on Traumatic Brain Injury Through Suppressing NLRP3 Inflammasome
Source: Neurochem Res. 2023 Sep 13;49(1):184–98. doi: 10.1007/s11064-023-04021-8 (PMC10776743; doi:10.1007/s11064-023-04021-8)
Supplement: Supplementary file 1 — Supplementary Material 1 [file 11064_2023_4021_MOESM1_ESM.docx]

**Supplementary Materials**


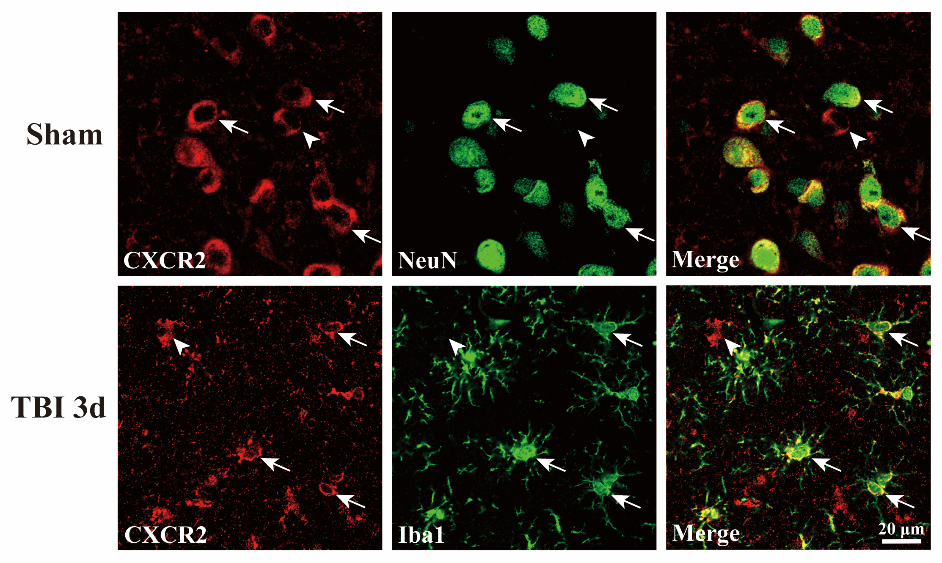


**Supplementary Fig. 1** Staining patterns of CXCR2 in the cortex of controls (Sham) and traumatized (TBI) mice. (Upper row) Double staining of CXCR2 (red) with neuronal marker NeuN (green) in cortical brain region of control tissues. Colocalization (arrows) is displayed in yellow, whereas arrowhead indicates lack of colocalization. (Bottom row) Double immunofluorescence staining of CXCR2 (red) with the microglia marker Iba1(green) in the peri-contusional cortex at 3 days post-TBI. Colocalization (yellow) can be observed in the overlay. Arrowhead shows devoid of colocalization (*n* = 5 per group).
